# Hippotherapy in the Treatment of CMD and Bruxism in Dentistry

**DOI:** 10.3390/ani15172587

**Published:** 2025-09-03

**Authors:** Margrit-Ann Geibel, Daniela Kildal, Amina Maria Geibel, Sibylle Ott

**Affiliations:** 1 Dento-Maxillofacial Radiology, Department of Maxillofacial Surgery, University Hospital Ulm, 89070 Ulm, Germany; 2 Department Gender-Specific Dentistry, Danube University, 3500 Krems an der Donau, Austria; 3 Department of Diagnostic and Interventional Radiology, University Hospital Ulm, 89070 Ulm, Germany; daniela.kildal@hopitalvs.ch; 4 Radiology, Upper Valais Hospital Center (SZO), Hôpital du Valais, 3900 Brig, Switzerland; 5 Department of General Pediatric Dentistry, University Center of Dentistry Basel, 4058 Basel, Switzerland; 6 i3R Center, Ulm University, 89081 Ulm, Germany; sibylle.ott@uni-ulm.de; 7 Endocrinology and Physiology, University Ulm, 89081 Ulm, Germany

**Keywords:** animal welfare, stress signals, stress reduction, horse assisted therapy, equine therapy, animal assisted therapy, hippotherapy, HRV (heart rate variability), CMD (craniomandibular dysfunction), bruxism in dentistry, stress index

## Abstract

Dysfunctions and disorders of the craniomandibular system are accompanied by pathophysiological changes of muscle groups in the throat/neck and facial area, e.g., pain in the jaw and muscles of mastication and disturbance of occlusion, leading to teeth injury (loss of dental hard tissue, fractures/sensibility disorders, etc.). For muscular dysfunctions, even in the context of psychosomatic disorders and chronic stress, hippotherapy is particularly suitable, since it helps actively to relieve muscle tensions. In the current project we combined hippotherapy with progressive muscle relaxation (PMR) to achieve a synergistic effect. The horses used for therapy (two mares and five geldings between seven and twenty-one years old) were especially suitable because of their calm temperament. In two cases, trained therapy horses were used; in five other cases, the patients used their own horses, which were not specially trained. Right from the beginning, the project was accompanied by veterinary support. Conditions of horse keeping (active stable, same-sex groups, no boxes) were assessed as well as the horses themselves prior to, during, and after each therapy unit. In patients, cortisol, as a quantifiable parameter for stress, was measured before and after each therapy unit. From before the start until the end of each therapy unit of 15 min, the heart rate variability (HRV) of both patients and horses was registered continuously and synchronously. In addition, the behavior of the horses was monitored and recorded on video by an experienced coach and a veterinarian. The stress load during the tension phases in the therapy units was low, perceivable in the horses lifting their heads and a slightly shortened stride length. Likewise, the horses reflected the patients’ relaxation phases, so that at the end of the units the horses were physically and psychically relaxed, too, noticeable by lowering their necks, free ear movement, and a decreasing heart frequency (HF). Altogether, the horses benefited from the treatment, too. Obvious stress signs like unrest, head tossing, tail swishing, or tense facial expressions were not noticed at any time. Twenty jumpers served as a control group in different situations (training, tournament, and leisure riding).

## Simple Summary

Dysfunctions and disorders of the craniomandibular system are widespread. For muscular dysfunctions, even in the context of psychosomatic disorders and chronic stress, hippotherapy is particularly suitable, since it helps actively to relieve muscle tensions. In the current study we combined hippotherapy with progressive muscle relaxation (PMR) to achieve a synergistic effect. In all cases a reduction of the stress levels could be monitored after the procedure. And the horses reflected the riders’ relaxation phases, so at the end both the horses and the riders were physically and psychically relaxed. In this way it opens the opportunity to have a fast procedure to reduce the stress level of the human-horse team before a start in a competition.

## 1. Introduction

Dysfunctions and disorders of the craniomandibular system may be accompanied by pathophysiological consequences typical for the human musculoskeletal system: discoordination of synergistic and antagonistic muscle groups, myalgias, muscle tensions, muscle gelling, muscle hypertrophy and hypotrophy, as well as primary jaw joint disorders, disc displacements, and other secondary pathological jaw joint changes.

Regarding the etiology of craniomandibular dysfunction (CMD), a multicausal disease is assumed, including micro traumas and macro traumas, constitutional conditions, hormonal disorders, bio-psycho-social factors, habits, and orthopedic as well as occlusal dysfunctions in stasis and motion [1].

Because of the proven participation of a discoordination of muscle groups and of muscle hypertrophies and hypotrophies, hippotherapy is predestined for a preventive but also for an accompanying therapy of this disease [2].

### 1.1. Current State in the Treatment of CMD

Treatment is indicated for patients with pain symptoms or functional limitations and is now implemented by dental and medical procedures [3]. The basic principle consists in the step-by-step recording of different pathophysiological findings in functional diagnostics in order to choose suitable therapies on this basis. Neoplastic and similar diseases should be clarified through differential diagnosis, and, if necessary, patients should be referred to special treatment early before starting a dental therapy.

### 1.2. Current State of CMD Hippotherapy

In dentistry, an interdisciplinary approach to treating CMD has been pursued successfully for some time. Occlusal and physical medical treatments are common. Since mastication system dysfunctions include somatic causes, especially the psychogenic partial causes, which are stress-induced, they are excellently suitable for treatment in the context of hippotherapy. In this field, no scientific research studying the proven positive effect of and experience from hippotherapy for muscle relaxation and stress reduction for this progressing CMD syndrome has been undertaken so far.

So far, hippotherapy has not been included in CMD treatment.

Yet the deeply relaxing and curative effects on the whole organism are undoubted and well proven [4]. Horse riding and ties with horses have a gender-sensitive aspect. More girls and women are attracted to the intensive interaction with a horse. Our own studies prove a gender-sensitive aspect of CMD. Hence, more female patients seem to contract CMD.

### 1.3. Present Therapeutical Concept for Bruxism/CMD

In patients with chronic and lengthy, therapy-resistant courses, the presence of a psychical comorbidity (e.g., depression, somatoform pain disorder, or personality disorder) or a severe or chronic psychosocial stress situation, respectively, should be clarified at the time of the first appearance of complaints or exacerbation [2,5].

In cooperation with a specialist for psychosomatic medicine and psychotherapy or a psychiatrist and psychotherapist, respectively, or a relevant and experienced psychologist, a wide range of psychotherapeutic treatments (psychodynamic or behavioral therapy, biofeedback, PMR, yoga, autogenic training, etc.), depending on the diagnosis, should be provided. Other therapies, such as acupuncture, acupressure, or transcutaneous electrical nerve stimulation (TENS), can be applied to normalize muscle function successfully or reduce muscle-induced pain [2].

### 1.4. Associated Clinical Measures Within the Multi-Disciplinary Therapy of Bruxism/CMD

The patient should be made aware of parafunctions and incorrect postures, e.g., through education and instruction in self-observation. The suspicion of psychogenic (‘stress-related’) partial causes of a chronic functional or pain disorder should be discussed with the patient [2]. All patients/participants in our research study were female. The members of the intervention groups received standardized information about the origin of bruxism and a DVD for a home training program to continue therapy beyond the single treatment appointments with relaxation training by PMR.

Massages and other physiotherapeutic treatments (e.g., chiro-therapy with impact on muscles and jaw joints, including osteopathic techniques and isometric tension and isotonic movement exercises) were researched for CMD treatment. As a home training program, these exercises enable therapy beyond the single treatment appointments [2].

### 1.5. Goal of the Study

Based on the positive results of research into Progressive Muscle Relaxation by Jacobson in the treatment of stress, anxiety, and tension [6,7], a scientific pilot project combined PMR with hippotherapy to investigate its potential effectiveness in treating the multifactorial condition of CMD.

For this purpose, various stress measurements were taken:

### 1.6. Cortisol Measurement

There is a circadian rhythmicity of cortisol. Immediately before waking up, the salivary cortisol level rises and peaks at about 30 min after awakening. This rise by ideally 60% is called cortisol awakening response (CAR) and proves the regenerative capacity of the hypothalamic-pituitary-adrenocortical system (HPA system). One hour after awakening, the cortisol level has already decreased sharply and continues to decrease throughout the rest of the day.

Stress induces the release of cortisol in the body from the adrenal cortex. A long-term risk, among others, is the significant and dose-dependent decrease in bone tissue (osteoporosis) going along with an increased fracture risk and poorer fracture healing [8,9,10,11].

Stress loads can be analyzed based on laboratory parameters to find out their causes and determine possible therapies. Moreover, in mental illness in particular, it is not only the physiological stress response that promotes the disease process but also a dysregulation of the neuroendocrine system (often induced by chronic activation). Salivary cortisol parameters have been used successfully for this purpose for years [12,13,14]. In studies by Pilger et al. from 2018 [15], the traditional approach of saliva sampling is questioned, and a reduction of samples is suggested, yet obtaining reliable results for stress research [15].

### 1.7. HRV Measurement

HRV measurement is becoming increasingly popular in stress research as an alternative to the long-established cortisol saliva test. Every human being shows a unique, personal HRV. The Boston-based fitness wearables company Whoop has compiled statistics showing the mean HRV by age group, which are classified as follows (see Table 1):

The HRV varies throughout the day. In addition, it may differ from day to day [16].

HRV measurements represent the interaction between the sympathetic and the parasympathetic nervous systems. This interaction maintains the homeostasis of physiological excitation [17,18]. HRV measurement is one of the best objective measurement values regarding regeneration and thus stress tolerance and performance of the body. The higher the variability of the heart frequency, the higher the readiness of the body to perform highly [19].

Already in 1963, HON and LEE realized the clinical relevance of the heart rate variability (HRV). Disorders of the fetal general condition caused changed intervals of consecutive heartbeats before changes of the heart frequency were noticed. In the 1980s, HRV was recognized as a significant prognostic mortality indicator after acute myocardial infarction [20,21]. In further studies, HRV measurements were significantly associated with stress [22].

The psychometric instrument State-Trait Anxiety Inventory was most frequently used in the studies as a comparison tool for HRV measurement.

In summary, it can be said that HRV measurement represents a valid parameter for psychic stress reaction.

HRV is registered via RR intervals. In the process, the distances between two heartbeats—two R waves—are analyzed. The term NN interval (normal to normal) is used as a synonym. Various statistical parameters of heart rate variability can be derived from the NN interval data.

The standard deviation of NN intervals (SDNN) is an indicator for the level of the overall variability. High SDNN values correlate with high heart rate variability and vice versa. Another statistically frequently used parameter is the root mean square of successive differences (RMSSD). It provides information about the activity of the parasympathetic nervous system. The rule of thumb is, “In a 24 h long-term measurement, SDNN values between 50 and 100 milliseconds are considered normal. For RMSSD, values between 30 and 70 milliseconds are considered normal” [23].

### 1.8. Stress Measurements in a Horse

The normal pulse rate of a horse is about 30 to 40 beats per minute. A deviation of five beats per minute is normal [24]. In comparison, horses without special clinical and echocardiographic findings in the heart showed a longer mean NN interval (mean NN) in the morning and a higher mean heart rate p (mean NN) = 0.0592 and p (mean HR) = 0.06) in the afternoon. The RMSSD in horses without pre-existing cardiac disease (mean value 81 in the morning—85 in the afternoon in healthy horses) did not show any significant differences compared at different times of the day [25]. The findings of the present study suggest that—besides the factors described above; which have an impact on the HRV—a circadian rhythmicity might be momentous; which has already been described for humans [26]. The parasympathetic system was dominant in the morning hours, as was the sympathetic nervous system in the afternoon [25,27,28].

### 1.9. Baevsky Stress Index (SI)

Professor Dr. Roman Markovich Baevsky played a key role in developing the method of variation pulsometry and defined, among other things, the Index of Regulatory Systems Tension. The latter is commonly referred to as the SI and is classified as an HRV parameter [29,30].

On the HRV parameter SI, short descriptions can be found that are different depending on the point of view from which they were made:

It is a commonly recognized measure for the characterization of recorded ECG signals or RR intervals.

It indicates the degree of central control of the heart rhythm and characterizes the activity of the sympathetic part of the vegetative nervous system (VNS). It is regarded as an indicator for shifts in the VNS balance and for changes in the balance between the impact of the sympathetic and the parasympathetic nervous systems.

The SI is also called the adjustment index of the regulation systems of the human organism. Professor Baevsky also uses the terms “Index of Regulation Strain” or “Straining Index” [29,30].

The SI is expressed in the form of a frequency distribution (histogram) of RR intervals measured. It describes the height-width ratio as well as the height (number)-to-width (variation) ratio of the histogram. A low number of similar RR intervals and a large number of different RR intervals (high variability) hint at sound HRV.

For comparison: a triathlete during endurance sports has an SI between 76 and 100 (high stress level) and 0–25 in the recovery phase.

## 2. Methods

The participants in this international study were female riders from Germany and Switzerland exclusively. The German female riders were recruited from the jaw-joint consultation hours at the Department of Oral and Maxillofacial Surgery at the Ulm University Hospital (ethics application 11/23, approved by the ethics commission of Ulm University). This study focuses on female riders because more female patients seem to contract CMD, and the majority of riders are female (e.g., in Germany, 76%). Parafunctions and false postures were made apparent to the patients’ minds, e.g., by education and instruction for self-observation. All participants in the intervention group received instructions on PMR by a certified PMR instructor and, in addition, a DVD with guided PMR exercises for home training, with the request to perform these at least once a day.

The aim was to investigate stress levels during riding using a standardized procedure developed by the German Society for Functional Therapy (Deutsche Gesellschaft für Funktionstherapie) for suspected CMD/bruxism, with the validated use of questionnaires (from medical psychology/bruxism questionnaires) [2]. Only female riders with suspected bruxism according to this validated screening by the DGZMKs professional association for functional therapy were included in the study. All test subjects were examined with MRI of the teeth if there were justifiable indications. The evaluation of the incidental findings on MRI of the temporomandibular joint was performed by the Department of Medical Radiology at Ulm University Hospital [31].

Intervention was made per participant throughout the whole observation period of six months.

At the first appointment (intervention 1), the patients in the intervention group were instructed on the exercises on a trained therapy horse. Interventions 2 and 3 were performed on the therapy horse or on a horse of their own. The control group (jumpers) rode on their own horses exclusively.

In both groups (intervention and control), saliva samples for cortisol measurement were taken early in the morning and 15 min after getting off the horse. The evaluation was carried out at the SwissHealthMed laboratory in Feldkirchen.

In the intervention group, the HRV was measured synchronically with female riders and horses using an HRV watch (by POLAR, Kempele, Finland). Measurements started immediately after mounting the horse and before starting the intervention. They were finished after the female riders dismounted their horses.

In parallel, video sequences were taken, and the animals’ behavior was observed by a coach and a veterinarian. The horses’ postures of necks and ears, their stride lengths, and their “snorting-off” were assessed as relevant behavioral parameters [32,33].

The horses in the intervention group were between seven and twenty-one years old and included five geldings and two mares. They were kept in same-sex groups in an active stable with free access to a lounging area and outdoor space and additional access to paddocks in summer. Hay was fed ad libitum, and concentrated feed as required. The horses in the control group were kept in paddock boxes with two hours of free roaming every day. They were between eight and fifteen years old and trained for the scheduled purpose (jumping).

### 2.1. HRV and HF Measurement

In addition to PMR, the HRV data of the participants and the horses in the intervention group were to be included in the study and evaluated as well prior to and during the three interventions. The data were recorded using a chest strap from Polar on the test person and the horse and recorded and evaluated using the validated Kubios standard version 3.5.0 software from Polar, Finland.

The pilot project for the treatment of CMD and bruxism in hippotherapy in Germany was scheduled with eight participants. The intervention group consisted of eight female riders who were instructed on a trained horse to perform PMR on their own and, furthermore, on their own horse.

During the three interventions (at the start, after three, and after six months), the HRV data of participants and horses were included in the study and evaluated. Furthermore, at all three appointments, salivary cortisol tests for the monitoring of possible stress reduction were made (in the mornings and 15 min after intervention).

One control group consisted of 15 semi-professional showjumpers from Switzerland who participated in 1 or more categories: Tournament, Jumping Instruction and Leisure Riding, resulting in 20 runs. In this group, only cortisol saliva tests (in the mornings and after riding) were evaluated. The female riders were examined during tournaments, riding instruction, and leisure riding over a period of four months.

The advantage of HRV measurements is their immediate availability during the interventions. There is no temporal delay through analyses in a lab taking several days. In our pilot study, a comparison between HRV and established salivary cortisol measurement was to be examined.

### 2.2. Animal Protection Issues

According to literature, there is multiple proof that therapeutic riding has no [34] or only little negative impact [32] on the horses used.

## 3. Results and Findings

### 3.1. Salivary Cortisol Measurement

The formation and release of cortisol are essentially involved in neuroendocrine stress reactions. The release underlies the circadian rhythmicity. That means that the cortisol release is synchronized in the body through a biological rhythm of about 24 h. The highest secretion takes place at six o’clock in the morning; its low is at midnight. The adrenocorticotropin hormone (ACTH) is inhibited by glucocorticoids and stimulated by the adrenocorticotropin-releasing hormone (CRH) and arginine vasopressin (AVP) [12,13,14,35].

Samples were taken for the first time immediately after getting up (within 30 min) and for a second time after intervention (between 12:30 and 1:30 p.m.).

#### 3.1.1. Findings with Female Riders in the Intervention Group (Germany)

Table 2 shows the findings with the German female riders in all three interventions over an intervention period of six months. First intervention in March 2023, second intervention in June 2023, and third intervention in September 2023.

All participants showed a decrease in salivary cortisol values between a minimum of −11% up to a maximum of −98% related to the saliva sampled in the mornings just after getting up. After the first intervention, the decrease was −51% to −98%. No difference could be found between the female riders doing their PMR exercises on a therapy horse and those riding on the horse of their own without doing any special exercises (−51% to −93%).

After the second intervention, the values with female riders from the group doing PMR on their own horse were −40% to −87%, and those of the female riders riding on their own horse but not doing PMR were −11% to −80%.

The female riders without PMR in intervention one and two did PMR in the third intervention on the therapy horse (one female rider was missing). In this intervention with the therapy horse, the decrease was between −61% and −65%. Riding the therapy horse and doing PMR exercises seems to have resulted in decreasing cortisol values in the three female riders.

In the third intervention, the female riders having carried out PMR until then (participants one to three) had to perform a riding instruction. In that intervention, the cortisol decrease was lower, at −26% to −55% (previous interventions one and two: −40% to −98%). Since the intervention group was very small, merely the different female riders’ individual stress-reduction profiles should be considered, in general.

Nonetheless, it can be stated that the interaction with horses results in a decrease in salivary cortisol values.

#### 3.1.2. Findings with Female Riders in the Control Group (Switzerland)

The control group’s cortisol values were measured in training situations: tournament, jumping, and leisure riding. The results are shown in Table 3, Table 4 and Table 5.

The decrease in cortisol values at the tournament was generally between −3% and −98%. Since the female riders ran at different times at the tournament, the participants’ individual second values are indicated as cortisol underlies the circadian rhythmicity.

The decrease in cortisol values with the five female riders after the tournament was between −1% and −36%; during leisure riding, it was between −13% and −60% (see Table 5).

### 3.2. HRV Measurement Findings in Intervention Group Germany

HRV is the variation in time between consecutive heartbeats. The HRV value reflects the balance between the sympathetic and parasympathetic nervous systems. A high HRV therefore means that the body responds to both influencing variables alike—the sympathetic and the parasympathetic nervous systems. A low HRV value means that one division of the autonomic nervous system is dominant.

The SI is a commonly recognized measure for the interpretation of recorded ECG signals or RR intervals, respectively. It represents the degree of central control of the heart rhythm and characterizes the activity of the sympathetic division of the vegetative nervous system (VNS). It is considered as an indicator for shifts in the VNS balance and for changes in the balance between impacts of sympathetic and parasympathetic, too [19].

In our pilot study, the HRV of the horse and that of the rider were recorded simultaneously during the intervention (Polar, Finland). The evaluation was carried out using the validated Kubios software from Polar, Finland. The graphs show the last 5 min of the 15 min intervention.

In order to classify the measured values, a comparison with high-performance sports shall be made: a triathlete has a stress index of between 76 and 100 (high stress level) during endurance sports and 0 and 25 during recovery [19]. Fluctuations in all the values measured here are well within the lower range of stress via HRV measurement in high-performance sports.

Clear stress parameters of the horses, such as restlessness, head tossing or tail swishing, or tense facial expressions, were not observed at any time.

#### 3.2.1. Evaluation of the 1st Intervention—Germany

**Figure 1:**
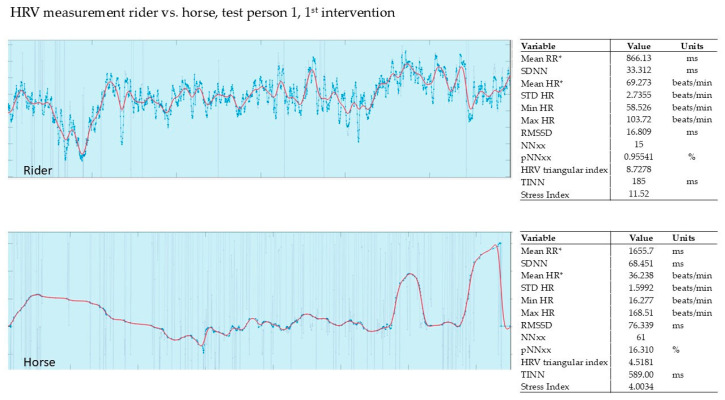
An example recording of the HRV of rider 1 during the first intervention. Red line shows HR (heart rate), blue line shows RR (time interval between successive ECG R-waves, RR interval; mean RR* and mean HR* are not calculated from the detrended RR interval data) [36].

The SI of the rider was determined to be max. 11.52 compared to the therapy horse with an SI of max. 4.0. Both rider and horse show a drop in HRV at the beginning. Towards the end of the intervention, both rider and horse show higher values. The HRV of the horse follows that of the rider with a delay. It is noticeable in the horse that tension and relaxation synchronize with the rider over the duration of the intervention with PMR. Especially at the end of the intervention, i.e., at the end of the recording, when all four muscle groups are being tensed (face, leg, and shoulder/neck muscles) and relaxed (see Figure 1).

#### 3.2.2. Evaluation of the 2nd Intervention—Germany—After Three Months

**Figure 2:**
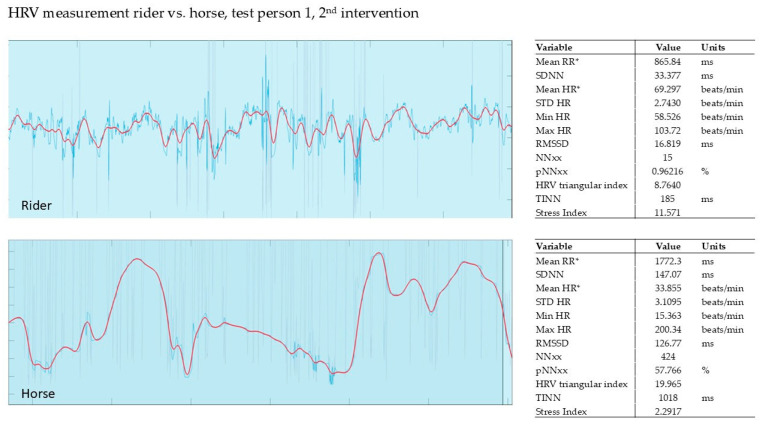
Rider 1, therapy horse, 2nd intervention, HRV. It shows the HRV measurement after 3 months. SI for the rider is 11.57; for the horse 2.2. Red line shows HR, blue line shows RR; mean RR* and mean HR* are not calculated from the detrended RR interval data.

Once again, synchronization of rider and horse can be observed during the exercises. This session could even document a ‘learning curve’ for rider and horse, as the rider and horse relax each other right from the start of the relaxation exercises (see Figure 2).

**Figure 3:**
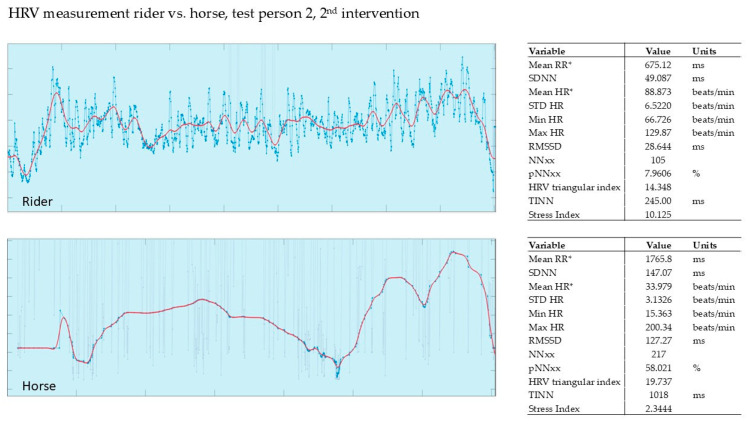
Rider 2, therapy horse, 2nd intervention, HRV. Red line shows HR, blue line shows RR; mean RR* and mean HR* are not calculated from the detrended RR interval data.

Figure 3 shows rider 2 during the 2nd intervention after 3 months. Rider 2 had an SI of 10.12, while the horse showed an SI of 2.3. The rider and horse are relatively synchronized, especially in the middle of the recording. The horse’s tension and relaxation follow the rider’s tension and relaxation peaks.

**Figure 4:**
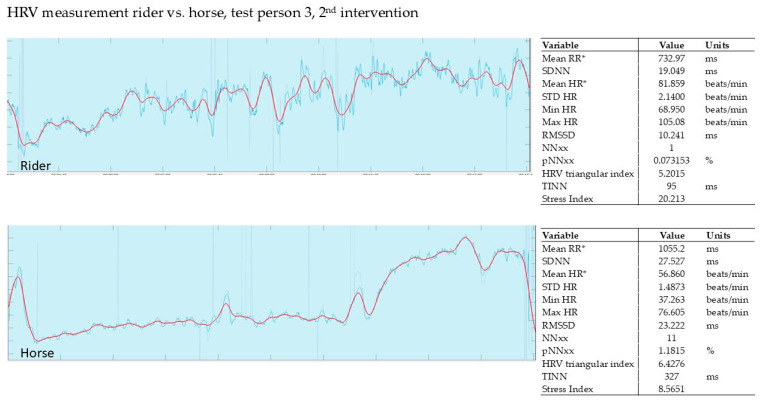
Rider 3, own horse, HRV. Red line shows HR, blue line shows RR; mean RR* and mean HR* are not calculated from the detrended RR interval data.

Figure 4 shows the recording of rider 3 during the 2nd intervention. The rider’s SI was relatively high at 20.2. The horse’s SI of 8.5 was also quite high compared to the therapy horses. One possible explanation could be that this horse is still being familiarized with the PMR exercises.

#### 3.2.3. Evaluation of the 3rd Intervention—Germany—After Six Months

**Figure 5:**
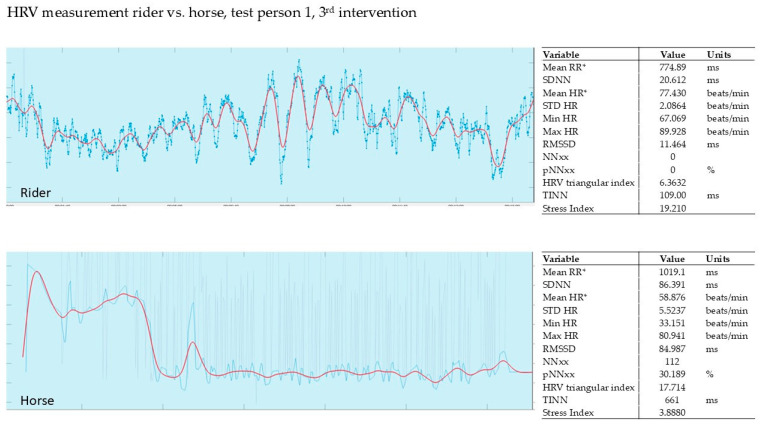
Rider 1, therapy horse, 3rd intervention. Red line shows HR, blue line shows RR; mean RR* and mean HR* are not calculated from the detrended RR interval data.

Figure 5 shows the HRV recording of rider 1 during the 3rd intervention. The rider’s stress index was 19.2, and the horse’s stress index was 3.8. The rider and horse were able to synchronize right from the start.

**Figure 6:**
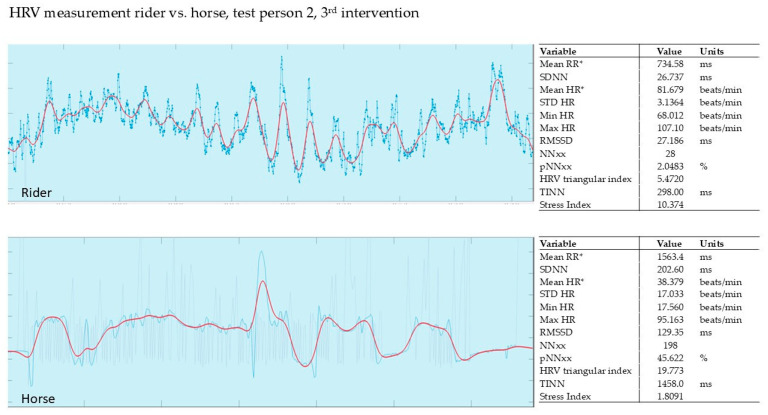
Rider 2, therapy horse, HRV. Red line shows HR, blue line shows RR; mean RR* and mean HR* are not calculated from the detrended RR interval data.

Figure 6 shows the HRV measurement of the 2nd rider during the 3rd intervention. The stress index of rider 2 was 10.3, while the stress index of the therapy horse was only 1.8. The horse adapted strongly to the rider. On average, both were more relaxed during the exercise than at the beginning or end of the exercise.

In this HRV measurement, the SI in the rider was noticeably low at max. 1.89 compared to the therapy horse at max. 2.11.

**Figure 7:**
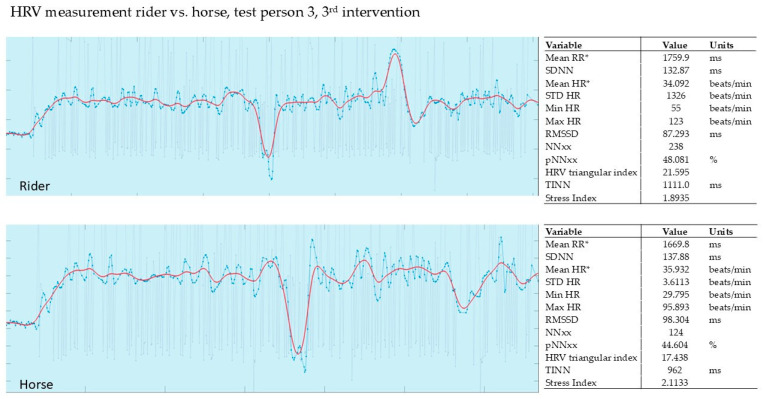
Rider 3, therapy horse, 3rd intervention. Red line shows HR, blue line shows RR; mean RR* and mean HR* are not calculated from the detrended RR interval data.

In the horse, it was noticeable that tension and relaxation were synchronized with the rider over the duration of the intervention with PMR. The muscle tension and relaxation phases were synchronized. Figure 7 shows a rider who was very experienced in PMR.

Parallel to the HRV measurements, the horses’ behavior was observed live and recorded on video synchronously with the measurements. During the tense phases, not only did the HRV increase, but the horses also shortened their stride lengths, their heads were slightly raised, and their ears turned backwards. The ears were held loosely or moved, and the horses snorted in a relaxed manner. Yawning, licking, and chewing could be observed towards the end of the therapy sessions in all the horses used.

## 4. Discussion

### 4.1. Stress Measurement

In our pilot study, the measurement of salivary cortisol, which is commonly used in stress research, was carried out in the intervention group in Germany together with the HRV measurement for the riders and the horse, synchronically with the HRV measurement. What is striking is the observed synchronization of rider and horse within 15 min of PMR under the guidance of the PMR coach.

With our pilot study, we were able to prove that HRV measurement is also suitable for observing stress reduction. Our graphs always show the last five minutes of the 15 min intervention. The horses clearly synchronized with their riders, both in terms of tension and relaxation. However, we would like to see further studies with more test subjects that evaluate both parameters and include the horses’ salivary cortisol values in the study.

The reduction in cortisol levels after the jumping instruction was between −1% and −36%; in leisure riding, between −13% and −60%. The reduction in cortisol values at tournaments was generally between −3% and −98%. It was surprising that, contrary to the assumption that a tournament would be stressful, seven out of eleven riders had a stress reduction of more than 20%. Four out of eleven riders even had a cortisol value of more than 50%. Here, too, a high individual cortisol value could be observed, which fits in with the established use of cortisol stress laboratory tests and the subsequent common practice of individual analysis.

In contrast, leisure riding in the four participating Swiss jumping riders appears to be less “stressful” than the jumping instruction. This result could be useful prospectively for an individual training plan for the riders.

All participating riders benefitted from handling the horse.

In this context, it was a surprising result that the reduction in stress measured in human saliva when interacting with horses was significantly higher than when interacting with dogs (maximum −98% for horses/maximum −20% for dogs). This provides a useful basis for future research [33,37,38].

If 80% of riders in Germany are female with an average age of 45, the reduction of a (chronically) elevated cortisol level, which is a risk factor for osteoporosis, is highly interesting.

In all groups the cortisol level of the riders decreased (mean −16% … −72%) by only one session. However, the SD (13.0% … 31.1%) shows that individual circumstances have a main influence on the level of stress reduction.

Due to the complexity and diversity of CMD and bruxism, an interdisciplinary approach is currently recommended. Depending on the type and nature of the CMD, this may include dentists (especially with a focus on functional diagnostics), physiotherapists, psychotherapists, orthopedists, pain therapists, doctors of manual medicine, ear, nose, and throat doctors, and speech therapists.

All parties involved must be aware that the therapy is usually symptomatic, which promotes and supports recovery and which commonly serves to relieve pain in the first place. It is not meant to be causal treatment, as is the case with caries therapy or root canal treatment, for example. This places an additional burden on the patient, which can have a negative effect on the symptoms [39].

Riding therapy has not yet been included in the treatment of CMD. However, the proven deep relaxing and healing effect on the entire organism is undisputed and sufficiently proven. Riding and the relationship with the horse have a gender-sensitive aspect. More girls and women feel drawn to the intensive interaction with the horse. Our own studies show a gender-sensitive aspect in CMD [39]. Our pilot study also showed an increased presence of psychological stress factors. None of the riders in the intervention group were free from any indications after evaluation of the validated questionnaire (DAAS) of the DGFDT [2].

In our pilot study, we were unable to detect any improvement in the clinical picture of bruxism over the observation period of six months. Therefore, the observation period was extended to one year after the pilot study as part of a planned follow-up study. However, we did observe a reduction in stress among the test subjects when handling the horses.

### 4.2. Aspects of Animal Welfare

It has been repeatedly documented in the literature that therapeutic riding has no [34] or only minor negative effects [32] on the horses used. A certain amount of stress on therapy horses is only reported when working with socially conspicuous children [32]. Various authors have observed greater stress on the horses due to the influence of experienced riders than due to hippotherapy [32,40]. We were unable to confirm this effect.

It has been noted several times that not only the patients but also the horses relaxed during the therapy sessions [41,42], which corresponds to our results. Both patients and horses were visibly relaxed at the end of the therapy sessions. However, we were also able to show that horses mirror their riders not only in their relaxation but also in their tension, i.e., there is a fundamental risk of stress.

‘Hippotherapy’ is not a precisely defined term. The use of therapy horses ranges from mental illnesses such as PTS (war veterans) via physical ailments down to social therapy in the context of social disorders or rather for general ‘well-being’. In principle, any type of use can be stressful for the horses, as their resilience and willingness to relax depend on their welfare and care conditions, the training, the behavior of the subjects, and the quality of the people carrying out the work. Just like ‘hippotherapy’, ‘hippotherapist’ is not a protected term; the level of training of coaches/supervisors varies greatly [43].

Signs of stress in horses include restlessness, tail swishing, holding the tail to the side, shaking the head, holding the head high with a tense lower neck, tense irregular movements, and even breaking out and actively defending themselves. None of the horses used here showed any signs of stress, neither in their HF/HRV nor in their behavior. However, the horses mirrored the riders’ phases of tension with slightly elevated HRV, shortened stride lengths, slightly raised heads, and ears turned backward in some cases. During the riders’ relaxation phases and particularly at the end, the horses lowered their necks, kept their ears relaxed or moved them, and snorted. Yawning, licking, and chewing off were also observed in all horses used.

We conclude that the use of horses for this form of therapy is not detrimental when carried out correctly. On the contrary, the horses could even benefit from the relaxation they experience. However, this requires the therapists to have appropriate expertise in horse training and horse behavior.

However, there are currently no generally accepted guidelines for animal welfare in animal-assisted therapy [33], even though some approaches already exist [33,44,45,46,47]. Therefore, improving the data available and developing general guidelines would be desirable.

Since physical and medical measures generally have a symptomatic effect and thus serve to quickly relieve pain, their use should be considered particularly in initial therapy but also in chronic cases. Prerequisites for this are an accurate diagnosis, proper instruction, careful implementation on the patient, and coordination with the treating dentist [2].

## 5. Conclusions

Due to the short observation period, we found no significant effect on CMD.

Our study results show a reduction in stress when handling horses, both in recreational riding, competitive riding, and during PMR training sessions. Both professional and recreational riders might benefit from reducing their stress level before competition.

Horses synchronize significantly with their riders, both in tension and relaxation.

The findings obtained from the pilot study form the basis for follow-up studies already scheduled in Austria and Switzerland with a preventive approach for riders suffering from bruxism/CMD. The follow-up period should then be significantly extended to a minimum of one year.

## Figures and Tables

**Table 1:** HRV standard values by age.

Age Group	HRV Standard Values
20 to 25 years	HRV 55 to 105
30 to 40 years	HRV 38 to 80
50 to 55 years	HRV 30 to 55
60 to 65 years	HRV 25 to 45

**Table 2:** Survey of salivary cortisol values [pg/mL] of all three interventions. The first valuewas measured in the early morning, the second immediately after intervention. The table shows mean values and SD (standard deviation) of the reduction.

	1st Intervention T1		Δ	2nd Intervention T2		Δ	3rd Intervention T3		Δ
Participant No.	1–1	1–2		2–1	2–2		3–1	3–3	
1	8.28	2.44	−71%	5.47	3.26	−40%	10.40	5.48	−47%
2	8.91	0.19	−98%	18.60	2.63	−86%	9.91	4.41	−55%
3	13.40	5.63	−58%	10.10	1.35	−87%	2.66	1.98	−26%
4	7.62	2.81	−63%	3.63	2.00	−45%	9.72	3.79	−61%
5	7.45	0.54	−93%	9.32	1.83	−80%	*	*	*
6	10.80	3.54	−67%	3.06	2.73	−11%	13.20	4.43	−66%
7	5.19	2.53	−51%	8.75	3.00	−66%	9.90	3.50	−65%
SD			17.6%			28.5%			15.1% *
mean			−72%			−59%			−53% *

Notes: All participants: riding + PMR. * Participant No. 5 could not keep the appointment of her 3rd Intervention T3.

**Table 3:** Salivary cortisol findings [pg/mL] with Swiss jumpers measured in the morning (T1) and after the tournament (T2).

Control Group Jumpers Switzerland					
Tournament					
Participant No.	1st Sample	2nd Sample	T1	T2	Δ
1	6:10	18:40	4.80	3.47	−28%
2	5:15	13:00	5.86	0.14	−98%
3	5:50	12:50	15.80	7.41	−53%
4	5:35	13:00	12.60	12.20	−3%
5	7:00	17:40	11.30	9.36	−17%
6	6:40	17:40	11.40	1.47	−87%
9	7:00	14:00	5.48	2.33	−57%
10	4:21	14:57	2.97	1.89	−36%
11	6:30	15:30	15.40	9.70	−37%
SD					31.1%
mean					−46%

**Table 4:** Salivary cortisol findings [pg/mL] with the control group (jumping instruction) measured in the morning (T1) and after riding (T2).

Control Group Jumpers Switzerland					
Jumping Instruction					
Participant No.	1st Sample	2nd Sample	T1	T2	Δ
1	13:00	14:00	6.86	4.39	−36%
2	7:30	18:30	6.83	6.04	−12%
3	6:00	18:30	9.87	8.02	−19%
4	8:00	18:30	14.80	14.70	−1%
5	7:00	19:00	2.98	2.65	−11%
SD					13.0%
mean					−16%

**Table 5:** Control group (leisure riding), cortisol measurement [pg/mL] in the morning (T1) and after riding (T2).

Control Group Jumpers Switzerland					
Leisure Riding					
Participant No.	1st Sample	2nd Sample	T1	T2	Δ
1	7:00	11:30	15.50	12.80	−17%
2	7:00	13:00	9.94	8.69	−13%
3	9:00	17:00	6.40	4.43	−31%
4	7:30	17:00	13.20	5.29	−60%
SD					21.3%
mean					−30%

## Data Availability

Data supporting reported results and conclusions of this article can be made available by the authors on request.
